# Artificial intelligence-driven genotype–epigenotype–phenotype approaches to resolve challenges in syndrome diagnostics

**DOI:** 10.1016/j.ebiom.2025.105677

**Published:** 2025-04-24

**Authors:** Christopher C.Y. Mak, Hannah Klinkhammer, Sanaa Choufani, Nikola Reko, Angela K. Christman, Elise Pisan, Martin M.C. Chui, Mianne Lee, Fiona Leduc, Jennifer C. Dempsey, Pedro A. Sanchez-Lara, Hannah M. Bombei, John A. Bernat, Laurence Faivre, Frederic Tran Mau-Them, Irene Valenzuela Palafoll, Natalie Canham, Ajoy Sarkar, Yuri A. Zarate, Bert Callewaert, Ewelina Bukowska-Olech, Aleksander Jamsheer, Andreas Zankl, Marjolaine Willems, Laura Duncan, Bertrand Isidor, Benjamin Cogne, Odile Boute, Clémence Vanlerberghe, Alice Goldenberg, Elliot Stolerman, Karen J. Low, Vianney Gilard, Jeanne Amiel, Angela E. Lin, Christopher T. Gordon, Dan Doherty, Peter M. Krawitz, Rosanna Weksberg, Tzung-Chien Hsieh, Brian H.Y. Chung

**Affiliations:** a Department of Paediatrics and Adolescent Medicine, School of Clinical Medicine, The University of Hong Kong, Hong Kong SAR, China; b Institute for Genomic Statistics and Bioinformatics, University Hospital Bonn, Rheinische Friedrich-Wilhelms-Universität Bonn, Bonn, Germany; c Institute for Medical Biometry, Informatics and Epidemiology, University Hospital Bonn, Rheinische Friedrich-Wilhelms-Universität Bonn, Bonn, Germany; d Genetics and Genome Biology Program, Research Institute, The Hospital for Sick Children, Toronto, ON, M5G 1X8, Canada; e Department of Pediatrics, University of Washington, Seattle, WA, 98195, USA; f Laboratory of Embryology and Genetics of Human Malformations, Institut National de la Santé et de la Recherche Médicale (INSERM) UMR 1163, Institut Imagine, Université Paris Cité, Paris, 75015, France; g CHU Lille, Centre de Référence Anomalies du Développement et Syndromes Malformatifs, Lille, F-59000, France; h Department of Pediatrics, Cedars-Sinai Medical Center, Los Angeles, CA, USA; i Division of Medical Genetics and Genomics, Stead Family Department of Pediatrics, University of Iowa Hospitals, Iowa City, IA, USA; j Centre de Génétique et Centre de Référence Anomalies du Développement et Syndromes Malformatifs, FHU TRANSLAD, Institut GIMI, Hôpital d’Enfants, CHU Dijon-Bourgogne, Dijon, France; k Equipe GAD INSERM UMR1231, Université de Bourgogne Franche Comté, Dijon, France; l UF 6254 Innovation en diagnostic Génomique des Maladies Rares, Centre Hospitalier Universitaire de Dijon, Dijon, France; m Department of Clinical and Molecular Genetics, University Hospital Vall d'Hebron and Medicine Genetics Group, Valle Hebron Research Institute, Barcelona, Spain; n Liverpool Centre for Genomic Medicine, Liverpool Women's Hospital, Crown Street, Liverpool, UK; o Department of Clinical Genetics, Nottingham University Hospitals National Health Service Trust, Nottingham, NG5 1PB, UK; p Section of Genetics and Metabolism, University of Arkansas for Medical Sciences, Little Rock, AR, 72701, USA; q Division of Genetics and Metabolism, University of Kentucky, Lexington, KY, USA; r Center for Medical Genetics, Ghent University Hospital, Ghent, Belgium; s Department of Laboratory Diagnostics, Poznan University of Medical Sciences, Poznan, Poland; t Department of Medical Genetics, Poznan University of Medical Sciences, Poznan, Poland; u Department of Clinical Genetics, The Children's Hospital at Westmead, Sydney, Australia; v Faculty of Medicine and Health, The University of Sydney, Sydney, Australia; w Unité INSERM U 1051, Département de Génétique Médicale, CHRU de Montpellier, Montpellier, France; x Department of Pediatrics at Vanderbilt University Medical Center, Nashville, TN, USA; y Service de Génétique Médicale and L'institut du Thorax, CHU Nantes, Nantes Université, CNRS, INSERM, Nantes, France; z Medical Genetics Service, Nantes University Hospital Center, Nantes, France; aa Normandie Univ, UNIROUEN, Inserm U1245, CHU Rouen, Department of Genetics and Reference Center for Developmental Disorders, FHU G4 Génomique, Rouen, F-76000, France; ab Greenwood Genetic Center, SC, USA; ac Centre for Academic Child Health, Bristol Medical School, University of Bristol, UK; ad Department of Clinical Genetics, UHBW NHS Trust, Bristol, UK; ae Department of Pediatric Neurosurgery, Rouen University Hospital, Rouen, 76000, France; af Medical Genetics, Mass General for Children, Boston, MA, 02114, USA; ag Genetics and Genome Biology Program, Research Institute, The Hospital for Sick Children, Toronto, ON, M5G 1X8, Canada; ah Division of Clinical and Metabolic Genetics, Department of Pediatrics, The Hospital for Sick Children, University of Toronto, Toronto, ON, M5G 1X8, Canada; ai Department of Biomolecular Medicine, Ghent University, Ghent, Belgium; aj Garvan Institute of Medical Research, Sydney, Australia; ak Department of Pediatrics, Guerin Children's at Cedars Sinai Medical Center, Los Angeles, CA, USA; al Diagnostyka GENESIS, Center for Medical Genetics in Poznan, Poland

**Keywords:** Splitting, GestaltMatcher, Methylation, Support vector machine, MN1, MCTT

## Abstract

**Background:**

Decisions to split two or more phenotypic manifestations related to genetic variations within the same gene can be challenging, especially during the early stages of syndrome discovery. Genotype-based diagnostics with artificial intelligence (AI)-driven approaches using next-generation phenotyping (NGP) and DNA methylation (DNAm) can be utilized to expedite syndrome delineation within a single gene.

**Methods:**

We utilized an expanded cohort of 56 patients (22 previously unpublished individuals) with truncating variants in the *MN1* gene and attempted different methods to assess plausible strategies to objectively delineate phenotypic differences between the C-Terminal Truncation (CTT) and N-Terminal Truncation (NTT) groups. This involved transcriptomics analysis on available patient fibroblast samples and AI-assisted approaches, including a new statistical method of GestaltMatcher on facial photos and blood DNAm analysis using a support vector machine (SVM) model.

**Findings:**

RNA-seq analysis was unable to show a significant difference in transcript expression despite our previous hypothesis that NTT variants would induce nonsense mediated decay. DNAm analysis on nine blood DNA samples revealed an episignature for the CTT group. In parallel, the new statistical method of GestaltMatcher objectively distinguished the CTT and NTT groups with a low requirement for cohort number. Validation of this approach was performed on syndromes with known DNAm signatures of *SRCAP, SMARCA2 and ADNP* to demonstrate the effectiveness of this approach.

**Interpretation:**

We demonstrate the potential of using AI-based technologies to leverage genotype, phenotype and epigenetics data in facilitating splitting decisions in diagnosis of syndromes with minimal sample requirement.

**Funding:**

The specific funding of this article is provided in the acknowledgements section.

**Research in context**

**Evidence before this study**

In clinical diagnostics, a consistent challenge involves accurate and detailed disease classification of syndromes, especially when disease mechanisms are not fully delineated. Decisions on “splitting” or “lumping” the subgroups of manifestations are of profound importance for precise diagnosis to aid the best appropriate clinical management yet approaches to objectively determine such splitting and lumping is relatively unexplored. A pubmed search was performed, using the keywords “lumping” OR “splitting” OR “machine learning” OR “syndrome” OR “delineation”.

**Added value of this study**

We took advantage of recent advances in AI integrated with multi-omics, including genotype and epigenotype to develop a combined approach to expedite the process of syndrome delineation. Specifically, we developed a new statistical method based on GestaltMatcher for facial morphology analysis in complementary DNA methylation analysis also using machine learning algorithms such as Support Vector Machine. We demonstrated that these methods can objectively differentiate two subgroups of patients with a relatively small number of sample requirements for truncating variants in different regions of the *MN1* gene. We further validated the applicability of these methods through 3 other genes previously described to cause NDDs (*SMARCA2, SRCAP, and ADNP*).

**Implications of all the available evidence**

With rapid advances of AI and multi-omics technology, our study highlights the tremendous possibility and capability of their application in delineating syndromic disorders and to facilitate the understanding of the mechanism, diagnostics, improve splitting decisions, provide insight into similar syndromes, even further aiding the advancement of precision medicine. We propose a possible wider applicability of such combinative analysis in more accurately differentiating syndromes with similar phenotypes, which could provide a more effective means to investigate genotype–phenotype associations.

## Introduction

Neurodevelopmental disease often presents with multiple subgroups of manifestations despite the same genetic cause. Decisions on the demarcation of syndromes, i.e., to either integrate (lump) manifestations into one comprehensive disease unit (syndrome) or differentiate (split) them can be challenging.^1^ This adds complexity to the understanding of the syndrome when the discovery is previously undescribed and the molecular consequences of the causal gene variants are not yet known.

Recent advances in computer vision have enabled the classification of disorders with dysmorphic facial features by analysing a patient's frontal image using next-generation phenotyping (NGP) approaches,^2^, ^3^, ^4^, ^5^, ^6^, ^7^, ^8^, ^9^ and such NGP tools can be helpful to facilitate syndrome delineation by analysing facial gestalt distinct to specific disorders.^10^, ^11^, ^12^, ^13^, ^14^, ^15^, ^16^, ^17^, ^18^, ^19^, ^20^, ^21^, ^22^ In addition to confirming whether a facial gestalt is associated with a particular genetic disorder, DeepGestalt can classify five subtypes of Noonan syndrome^23^; however, it requires prior training and thus is difficult to apply to emerging ultrarare disorders. Another state-of-the-art NGP approach, GestaltMatcher,^2^ quantitates facial similarities in patients and performs a cluster analysis of clinical face phenotype space without the need to train a dataset for a particular disorder. Therefore, it is more generalizable for delineating or “splitting” syndromes. A specific approach to support the decision of lumping and splitting based on statistical evidence has not been established in the GestaltMatcher Software.

DNA methylation (DNAm) can also be used to detect distinct disease signatures, with more than 60 unique DNAm signatures described for neurodevelopmental disorders.^24^, ^25^, ^26^, ^27^, ^28^ DNAm signatures not only reveal the differentiation of syndromic disorders but they may be used to assess variants of uncertain significance in neurodevelopmental disorders.^25^^,^^29^ Because of its use in diagnostics, DNAm can stratify subgroups of syndromic phenotypes based on variation location or type with good accuracy and provides insight into the underlying molecular mechanisms.^29^, ^30^, ^31^, ^32^, ^33^, ^34^, ^35^, ^36^

Individuals affected by rare neurodevelopmental diseases typically require at least five years to receive a confirmed diagnosis,^37^ and novel syndromes often require additional time to gather sufficient data to establish genotype correlations. To expedite the process of delineating syndromes, we demonstrated the feasibility of artificial intelligence (AI)-driven approaches (DNAm and facial gestalt) to prioritise and streamline these curation techniques. As proof of concept, we used this method on an expanded cohort of individuals diagnosed with a recently described^38^ neurodevelopmental syndrome. This syndrome is associated with alterations in the *MN1* proto-oncogene and is characterized by distinctive craniofacial features and a unique pattern of brain malformation, including rhombencephalosynapsis (RES).^38^^,^^39^ *MN1* is comprised of two exons. We characterized 23 affected individuals in whom C-terminal truncation (CTT) variants in the last 55 bp of exon 1 or exon 2, thus predicted to cause escape from nonsense-mediated mRNA decay (NMD).^40^ This work resulted in the establishment of *MN1* C-terminal truncation (MCTT) syndrome.^41^ In our initial report, only three individuals were identified with de novo truncating variants outside of the hotspot for those with CTT. These patients and subsequent case reports demonstrated that the N-terminal truncation (NTT) variants upstream of the last 55 bp of exon 1 were associated with nonspecific facial characteristics, conductive hearing loss, and less severe neurodevelopmental features compared to MCTT patients (n = 7 published NTT patients).^42^^,^^43^ The results of a transcription analysis of CTT skin fibroblasts revealed evidence of escape from NMD and the MCTT phenotype was believed to be the result of this mechanism. Miyake et al. also demonstrated that CTT of MN1 was associated with increased protein stability, reduced cell proliferation, and enhanced MN1 aggregation in vitro.^39^ A gain-of-function effect was postulated based on these observations. The predicted increase in the fraction of intrinsically disordered regions in MN1 CTT protein further implies a possible mechanism of altered phase separation.^39^

**Fig. 1:**
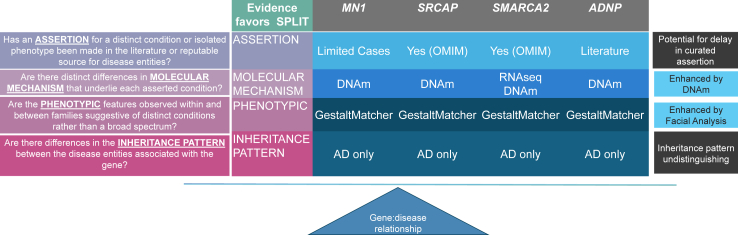
**Overview of combined GestaltMatcher and DNAm approach for splitting of gene–disease relationships according to ClinGen**. Evidence for *MN1, SRCAP, SMARCA2* and *ADNP* in relation to the ClinGen lumpers and splitters framework by Thaxton et al.^44^ The latter three are reported syndromes with DNAm by Rots et al.^30^ Chater-Diehl et al.^29^ and Bend et al.^45^ Our proposed approach enhances evidence for splitting my molecular mechanism (DNAm) and phenotypic features (GestaltMatcher).

In this expanded cohort of 56 individuals, we categorized subjects with *MN1* NTT and CTT variants and confirmed a distinct set of phenotypes in each group. In addition, we proposed an expanded GestaltMatcher statistical method and integrated 1) GestaltMatcher and 2) DNAm to facilitate objective assessments of the clustered subgroups based on facial gestalt and episignatures. This study represents the application of a combined NGP (GestaltMatcher) and DNAm approach to validate the previously observed pleiotropy associated with *MN1*, and to validate the effectiveness of GestaltMatcher in differentiating syndromic subgroups of *SRCAP*, *SMARCA2,* and *ADNP* with known distinct DNAm signatures (Fig. 1). We demonstrated that GestaltMatcher did not require a training dataset in the model involving several photos of target disorders, whereas it differentiated ultrarare disorders with only three patients per group. Moreover, a distinct DNAm signature for MCTT served as an alternative diagnostic tool to obtain results in concordance with those of GestaltMatcher. The flexibility and scalability of this method will contribute to the decision of splitting with a limited number of patients, thereby enhancing curation that adheres to the ClinGen lumping and splitting framework.^44^ In this expanded cohort of 56 individuals diagnosed with *MN1* truncating variants, we demonstrate that AI-driven approaches can be utilised to help delineate syndromic subgroups within the same gene and can be utilised to better inform splitting decisions in the early stages of syndrome delineation.

## Methods

### Patient recruitment and data from literature

Twenty-two unpublished individuals (C04, C05, C07, C09, C10, C11, C19, C21, C32, C33, C34, C35, C40, C41, C42, C44, N03, N06, N08, N09, N12, N13) diagnosed with truncating variants of the *MN1* gene were recruited from 20 independent research or diagnostic laboratories (Supplementary Fig. S1, Supplementary Table S1). Individuals from published studies of Mak et al.^41^ (C01, C06, C08, C12, C13, C14, C17, C20, C22, C23, C24, C25, C26, C27, C28, C29, C30, C36, C37, C38, C39, C43, N01, N04, N07), Tian et al.^46^ (C02), Zhao et al.^47^ (C03), Miyake et al.^39^ (C16, C18, C31), Vegas et al.^42^ (N02, N05) and Shu et al.^43^ (N10, N11) were also included in this expanded cohort analysis for photo facial gestalt analysis and/or clinical phenotype where available. A patient with a mosaic CTT variant (Individual 7) from the Mak et al. study^41^ was excluded. Our recruitment strategy focused on inclusivity, incorporating individuals from diverse racial and ethnic backgrounds to minimize bias in the study of the *MN1* gene-related disease.

### RNA sequencing

For RNA-seq analysis of fibroblasts from individuals N02, N13, and C38, total RNA was extracted using TRIzol and purified with an RNA Clean & Concentrator-5 kit (Zymo Research). mRNA library preparation (poly-A enrichment) and RNAseq were performed by Novogene. Sequencing of 150-bp paired-end reads on an Illumina NovaSeq instrument was performed. GATK's RNA-Seq best practices and the two-pass method of STAR (v2.5) were used to process the fastQ files and aligned them to the human genome. Duplicated reads were eliminated by Picard tools (v1.113). For RNAseq of fibroblasts from individuals C21, C22 and C33, the samples were lysed in TRIzolTM Reagent (Thermo Fisher Scientific, Waltham, Massachusetts, USA) for RNA extraction based on the manufacturer's instructions. The poly-A tail capture method with the KAPA mRNA HyperPrep Kit (Roche Diagnostics, Tucson, AZ, USA) was done to obtain a nonstrand-specific RNA library with an RNA integrity number (RIN) ≥8. RNA libraries were then processed as 150-bp paired-end runs on an Illumina NovaSeq 6000 platform (Illumina, San Diego, CA, USA) with a minimum of 100 million reads per sample at the Center for PanorOmic Sciences, University of Hong Kong. FastQ files were mapped to the hg19/GRCh37 human reference genome using STAR (v2.5.2a) in basic two-pass mode.

### GestaltMatcher clinical face phenotype space

GestaltMatcher is trained using 6354 frontal images of 5123 patients which encompassed 204 unique disorders from the GestaltMatcher Database (GMDB; https://db.gestaltmatcher.org/), with a specific focus on facial dysmorphic features. Each image was encoded by twelve 512-dimensional facial phenotype descriptors (FPDs) using GestaltMatcher, which ultimately spanned a clinical face phenotype space (CFPS) characterized by the FPDs. Facial similarities among patients were further quantified using the cosine distance between the respective FPDs.^2^^,^^48^ Patients within the CFPS can then be ranked based on the pairwise cosine distances.

### GestaltMatcher lumping and splitting approach (Comparison between two groups)

To validate whether two patient groups were similar or different from one another, we developed a statistical approach, the core of which was the estimation of their mean pairwise cosine distance. We selected 7459 images from 5995 different patients along with 449 different syndromes from the GMDB.^49^ We included those that had not been used in the training of GestaltMatcher^2^ and syndromes with at least two distinct patients. Finally, we obtained 1499 images from 1182 subjects and 321 syndromes for analysis. The syndromes were split randomly into five groups. The syndrome-based split of the dataset guarantees that multiple images of the same patient are in the same group, and training and test datasets are truly distinct. Using five-fold cross-validation, we included 4/5 of the syndromes for sampling two control distributions: (1) same syndrome distribution and (2) different syndrome distribution and derived a threshold *c* by ROC curve analysis to discriminate between same and different syndromes. The resulting threshold was applied for the assessment of a validation set (1/5 of the syndromes) and the fold with the highest validation metric was ultimately selected. The concept of building the same and different syndrome distributions is illustrated in Supplementary Fig. S2.

Using the ensemble model and test-time augmentation,^48^ each image *i* is represented in the CFPS as twelve 512-dimensional vectors $x_{i,k}\in R^{512},k\in \left[1,12\right]$. The distance d(i,j) is defined as the average of the twelve cosine distances between the images *i* and j, i.e., $d\left(i,j\right)≔\frac{1}{12}\sum_{k=1}^{12}\left(\;1-\frac{{x_{i,k}}^{T}x_{j,k}}{∥x_{i,k}{∥}_{2}∥x_{j,k}{∥}_{2}}\right)$.

For the same syndrome distribution and for each syndrome in our training set consisting of 4/5 of the syndromes, we randomly divided the associated images into two groups, $C_{1}$ and $C_{2}$. Then, we calculated the pairwise cosine distance d(i,j) between each image *i* from ${\;C}_{1}$ and each image j from the $C_{2}$, and computed the mean of those distances according to the following formula:

$$d\left(C_{1},C_{2}\right)≔\frac{1}{\left|C_{1}\left|\;\right|C_{2}\right|}\sum_{i\in C_{1},j\in C_{2}}d\left(i,j\right)$$

This was repeated 10 times per syndrome to provide a sampled distribution of mean pairwise distances between two groups connected with the same syndrome.

Regarding the different syndrome distributions, for each combination of two different syndromes in our dataset, we sampled one group with a random size from each of the two syndromes. Next, we calculated the mean pairwise cosine distance between the two groups. This was repeated five times for each combination of two different syndromes, thus enabling the determination of a sampled distribution of mean pairwise distances between two groups linked to different syndromes. If the same two groups were sampled twice (either from the same or from different syndromes), then the duplicated comparison was removed from the sampled distribution.

We conducted a ROC curve analysis of the sampled groups from the same syndrome (“controls”) and different syndromes (“cases”). The mean pairwise cosine distance was a measure of discrimination and yielded an area under the curve value of 0.94. We selected the threshold c to determine whether the two groups were similar (mean pairwise cosine distance < *c*) or different (mean pairwise cosine distance > *c*) by maximizing the Youden index^50^:

$$c=\arg\max_{c\in \left[0,1\right]}\;sensitivity\left(c\right)+specificity\left(c\right)-1$$

Notably, in this context, sensitivity refers to the proportion of true positives, i.e. two groups from different syndromes that were correctly split; specificity refers to the proportion of the true negatives, i.e., two groups from the same syndrome that were correctly lumped.

The same sampling schemes were applied in the validation set, which included the remaining 1/5 of the syndromes, and the Youden index corresponding to the threshold c was calculated. Finally, the fold corresponding to the highest Youden index in the validation set was selected, resulting in *c = 0.901,* which correlated with a sensitivity of 0.890 and a specificity of 0.851 on the training folds and a sensitivity of 0.911 and a specificity of 0.885 on the validation fold (Supplementary Table S2). To estimate the uncertainty in two groups, $C_{1}$ and $C_{2}$, we sampled 100 subgroups of random cohort size and calculated their mean pairwise cosine distance. If the proportion of those distances above the threshold c was greater than 50%, the analysis provided evidence for $C_{1}$ and $C_{2}$ stemming from different syndromes. To provide more insight into the distinctiveness of the cohorts, the positive predictive value (PPV) of two cohorts with a distance in the range of distances between the sampled subgroups of $C_{1\;}$ and $C_{2}$ (i.e., the probability of two cohorts with distance $d\in \left(\min\;d\left(C_{1}^{sub},C_{2}^{sub}\right),\max\;d\left(C_{1}^{sub},C_{2}^{sub}\right)\right)$ being truly distinct) was estimated based on the pooled control distributions constructed on the validation folds via

$$PPV=\frac{sensitivity*p}{sensitivity*p+\left(1-specificity\right)*\left(1-p\right)}$$

with *sensitivity* being the probability of a distance in the specified range for two cohorts stemming from different syndromes, *specificity* being the probability of a distance outside the range for two cohorts stemming from the same syndrome and *p* being the pre-test probability. We assumed a pre-test probability of 50% which reflects no prior information and the same probability of two cohorts stemming from the same or different syndromes.

### Lumping and splitting performance validation

We used the validation set of the selected fold to numerically represent the quality of our assessment by conducting two analyses:

1. We compared each combination of two distinct syndromes by calculating their mean pairwise cosine distance, sampling 100 subgroups, and determining the proportion of distances above the chosen threshold.
2. We split each syndrome randomly into two groups and conducted the same analysis as in step 1.

This analysis yielded a sensitivity (the proportion of true positive results) of 0.964 and a specificity of 0.773 (the proportion of true negative results) in the validation set.

### DNAm analysis

Whole blood DNA samples were subjected to bisulfite conversion using the QIAGEN EpiTect PLUS Bisulfite Kit and hybridization was done using the Illumina Infinium Human MethylationEPIC BeadChip, based on the manufacturer's guidelines. The *minfi* Bioconductor package in R was used to preprocess the raw DNAm array data, including normalization and background subtraction, as previously reported.^36^ After filtering, 639,563 probes remained, and beta (β) scores were calculated to display DNAm levels that were in a percentage range between 0 (absence of methylation) and 1 (completely methylated).

Differential DNAm analysis was conducted between nine patients with CTT variants and 41 controls using a regression model in the limma Bioconductor package. To account for potential confounding factors such as age, blood cell counts, and sex, principal components 1 and 2 (PC1 and PC2) were included as covariates in the model. These covariates helped to control for the variations associated with population structure and technical noise. Differentially methylated loci were identified by calculating moderated t-statistics, which provide more stable and reliable inference by borrowing information across genes and adjusting for small sample sizes. The resulting p-values were then adjusted for multiple testing using the Benjamini-Hochberg method, controlling the false discovery rate (FDR) to reduce the likelihood of type I errors (false positives). Differentially methylated sites were identified based on an FDR-corrected p-value <0.05 and a magnitude of Δβ >5%.

Visualization tools, including PCA and hierarchical clustering, were used to evaluate signature CpG sites and distinguish individuals with CTT variants from 41 age- and sex-matched control subjects. A SVM model was developed, trained with the differential methylation profiles for the discovery cases and controls at the signature sites, and generated SVM predictive scores between 0 and 1. Samples were classified as “CTT-like” (score >0.5) or “Not-CTT” (score <0.5). The specificity and sensitivity of the model were assessed using one additional CTT case, 79 additional controls as validation datasets, and five NTT variants for testing.

### Statistical analysis

The differences between clinical phenotypes of CTT and NTT groups were assessed using a two-tailed Fisher's exact test. Given the multiple comparisons, we adjusted the significance level using a Bonferroni corrected p-value of <0.0028 (0.05/18).

### Ethics approval and consent to participate

Genetic research was performed according to approved institutional ethical guidelines (Institutional Review Board of the University of Hong Kong/Hospital Authority Hong Kong West Cluster (HKU/HA HKW IRB) Ref number: UW 12–211). Informed consent for participation and consent to publish clinical photographs were obtained from all families.

### Role of funders

None of the sponsors had any role in the design and conduct of the study; collection, management, analysis and interpretation of the data; preparation, review, or approval of the manuscript; or in decision to submit the manuscript for publication.

## Results

### Expansion of the NTT and CTT subgroups of the *MN1* cohort

We expanded the number of samples for this clinical cohort (beyond 34 previously published cases) by recruiting 22 additional individuals exhibiting truncated variants of the *MN1* gene (Supplementary Fig. S1). We further analysed the difference in clinical features of the individuals affected by CTT (n = 43; 23 males and 20 females; 17 unpublished) and NTT (n = 13; nine males and four females; five unpublished) based on a boundary predicted by the 50–55 nucleotide rule for NMD^40^ at amino acid position 1244 (Supplementary Fig. S1). Clinical phenotyping followed by statistical analysis was performed on the 56 individuals to highlight the degree of difference in clinical characteristics between the NTT and CTT groups, including analysis of i) neurodevelopment, ii) neuroimaging, iii) hearing, iv) facial features (including cleft palate), and v) involvement of other systems (Supplementary Table S1; Supplementary Table S3, Supplementary Table S4, Table 1).

**Table 1:** Clinical features of 56 Individuals with *MN1* truncating variants from reported literature and this cohort.

Clinical features	CTT^a^	%	NTT^a^	%	p-value
RES (rhombencephalosynapsis)^e^	11/15	73	0/2	0	0.1103
Midface hypoplasia	27/36	75	0/8	0	0.0001^c^
Hypertelorism	34/36	94	1/8	13	<0.0001^c^
Skull shape anomalies	29/38	76	2/9	22	0.0042
Upper helix dysplasia	31/39	79	1/8	13	0.0007^c^
Downslanting palpebral fissures	25/32	78	3/8	38	0.0386
Intellectual disability	28/28	100	1/10	10	<0.0001^c^
Expressive speech delay	39/39 (Severe)	100	8/10 (Less Severe)	80	0.0383
Age of first words (in months)	50 (20–120)^b^		26 (18–36)^b^		
Only non-verbal communication	11/21	52	0/7	0	1
Hypotonia	32/34	94	3/9	33	0.0003^c^
Motor delay	40/42	95	1/10	10	<0.0001^c^
Age of independent walking (in months)	28 (18–60)^b^		No delay except N03 (delayed at 20 mo)		
Hearing loss (All)	28/35	80	10/12	83	1
Conductive hearing loss	10/35	29	10/12	83	0.0017^c^
Sensorineural hearing loss	6/35	17	0/12	0	1
Mixed hearing loss	3/35	9	0/12	0	0.5597
Unspecified hearing loss	9/35	26	0/12	0	0.087
Cleft palate	5/38	13	4/11	36	0.1786
High arched palate	25/38	66	2/11	18	0.0139^c^
Dental issues^d^	17/25	68	0/6	0	0.0041^c^
Feeding difficulties	24/38	63	6/11	55	0.7292
Obstructive sleep apnoea	3/9	30	1/3	33	1
Cardiovascular anomalies	18/33	55	1/9	11	0.0268^c^
Spinal anomalies	13/17	76	1/4	25	0.0877
Ophthalmological anomalies	23/33	70	1/9	11	0.0025^c^
Seizures	6/32	19	0/9	0	0.309

a Denominator denotes the number of individuals with phenotype information available.

b Mean age (range).

c Statistically significant with Bonferroni corrected p-value of 0.0028 (two tailed Fisher's Exact Test).

d Includes overcrowding of teeth, widely-spaced teeth, small/hypoplastic teeth, malocclusion, malposition, prognathism.

e Only frequencies of MRIs images available for review to confirm RES (Supplementary Table S4) are reported here.

Neurodevelopment evaluation revealed that there was a significantly higher number of individuals exhibiting CTT variants with intellectual disability (100% vs. 10%, p < 0.0001∗), hypotonia (94% vs. 33%, p = 0.0003∗), and motor delay (95% vs. 10%, p = <0.0001∗) (Table 1). Of note, the average degree of intellectual disability in the CTT groups is likely more severe than the case of NTT (N06) with moderate learning difficulties. In the NTT group, almost all achieved independent walking by 16 months (ie, normal) while the mean age for independent walking was 28 months for individuals with MCTT. Expressive speech delay was more profound in the CTT group, in which 52% (11/21) of the individuals relied on nonverbal communication only, whereas the remaining expressed their first words at a mean age of 50 months (range 20–120 months). In the NTT group, 80% exhibited a small degree of expressive speech delay with first words at a mean age of 26 months (range 18–36 months).

Brain MRI images were only available for 15 CTT cases for comprehensive analysis, including five in this extended cohort and ten previously reported cases.^38^ Eleven individuals (with MRI images for review as a series) presented with RES (Table 1, Supplementary Table S4). An additional four patients (C05, C19, C32 and C42) were also reported to have RES by clinical MRI report. Only two sets of MRI images were available for the NTT individuals, and no evidence of RES was observed in the limited available data (Supplementary Table S4).

With respect to hearing loss, patients with NTT variants showed a significantly higher incidence of conductive hearing loss (n = 10, 83%, normal in two), whereas both conductive (n = 10) and sensorineural hearing loss (n = 6) occurred in CTT patients (mixed type appearing in three individuals).

**Fig. 2:**
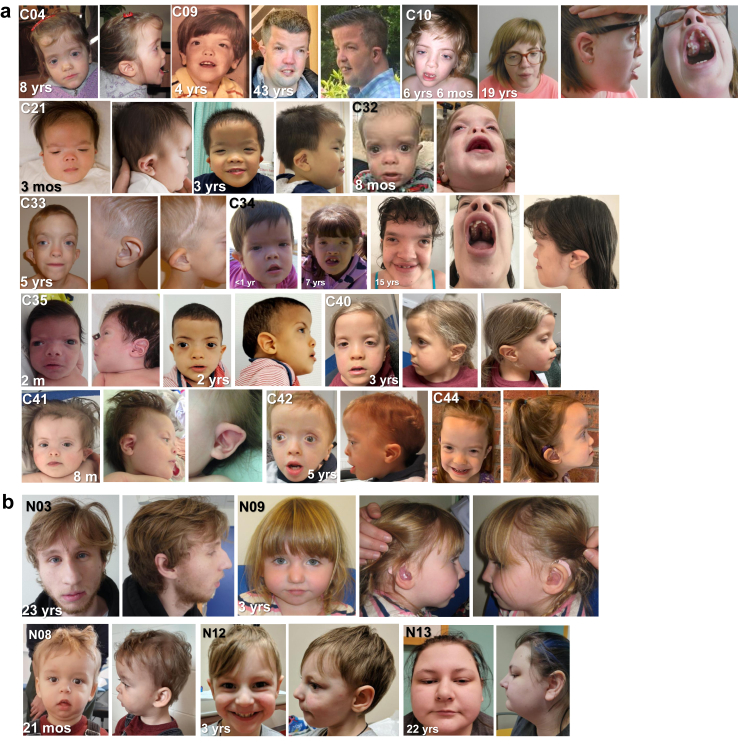
**a Clinical features of individuals with previously unreported *MN1* C-Terminal Truncations (CTT)**. The core recognizable facial features of MCTT (midface hypoplasia, downslanting palpebral fissures, hypertelorism, exophthalmia, short, upturned nose, and small low-set ear) are consistently observed. Individual C09 is the oldest known individual with MCTT to date. Oral photos demonstrate abnormal palate with thickened lateral palatine ridges and severe dental malposition and crowding (Individuals C10, C32, C34). **b Clinical features of individuals with previously unreported *MN1* N-Terminal Truncations (NTTs)**. Among all individuals with NTT, there is reduced height of the lower third of the face with micrognathia and the nose is often found to be prominent. Individual N03 with hypotelorism, long nose with prominent full tip, small mouth and microretrognathia; Individual N08 with high forehead, low nasal bridge, short nose, high arched palate and tented upper lip; Individual N09 with downslanting palpebral fissures and broad nasal bridge, and submucous cleft palate (not shown in photo); Individual N12 with upslanting palpebral fissures, high bridge of nose and micrognathia; Individual N13 (mother of N12) with broad tipped nose, long palpebral fissures and thin vermillion of the upper lip.

The assessment of facial features revealed a recognizable phenotype in the CTT group (Fig. 2a), which included midface hypoplasia, down slanting palpebral fissures, hypertelorism, exophthalmia, short upturned nose, and small low-set ears. The facial profile in the NTT group was much more subtle and challenging to recognize clinically as a distinct syndrome, yet NTT individuals were clearly distinct from the CTT gestalt. More consistent NTT features include reduced height of the lower third of the face with micrognathia, and the nose is often found to be prominent (Fig. 2b). Extending the results of Vegas et al.^42^ we observed a continued predominance of cleft palate in the NTT group (36% vs. 13%), whereas most of the other individuals with CTT (25/38, 66%) had a high-arched palate. With respect to other abnormalities involving other systems, a varying degree of feeding difficulties was observed in both groups. Cardiovascular (55% vs. 11%) and ophthalmological abnormalities (70% vs. 11%) were more prevalent in CTT (p < 0.05). Hence, the clinical observations in this extended cohort, in relation to i) neurodevelopment, ii) neuroimaging, iii) hearing, iv) facial features, and v) involvement of other systems, confirm our previous observations that the two *MN1* genotype groups can be readily distinguished by comparison of clinical features.

### **RNA-seq quantification of transcript expression**

**Fig. 3:**
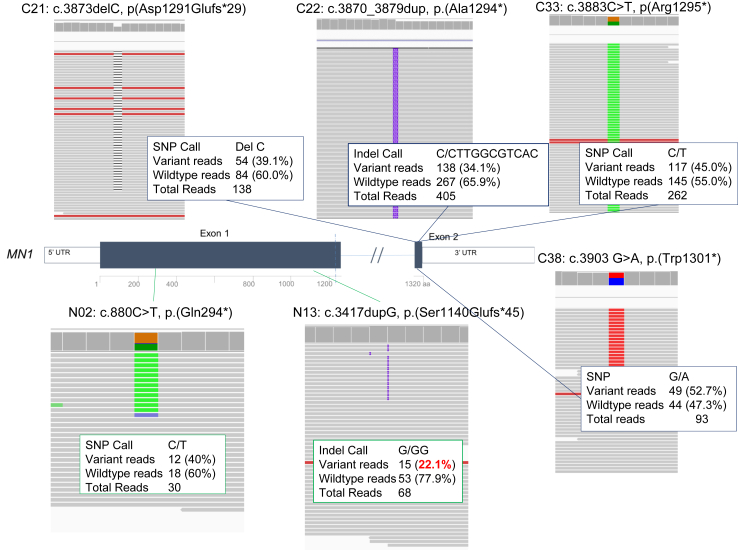
**RNAseq of NTT and CTT variants**. RNAseq data of fibroblast samples from two individuals (N02 and N13) with NTT variants and four individuals (C21, C22, C33 and C38) with previously reported CTT variants. Expression of NTT transcripts (c.880C > T and c.3417dupG) showed a read count of 40% and 22% respectively. Read counts for samples with CTT transcripts (c.3873delC, c.3870_3879dup, c.3883C > T, and c.3903G > A) were 39.1%, 34.1%, 45%, and 52.7% respectively. There was no significant difference between the expression of NTT and CTT transcripts.

The distinct phenotypes of the NTT and CTT groups are hypothesized to be due to haploinsufficiency for the former (either due to NMD, or in the case of NMD escape, non-functional protein due to extreme truncation), vs. gain of function for the latter. To explore the molecular basis of CTT and NTT at the transcript level, RNAseq was done in a limited number of samples. Because *MN1* expression in blood is undetectable, RNA-seq analysis was performed in fibroblasts. Fibroblast samples were available from six individuals (N02, N13, C21, C22, C33, and C38) with the NTT variants, c.880C > T and c.3417dupG, or the CTT variants, c.3873delC, c.3870_3879dup, c.3883C > T, and c.3903G > A. The NTT transcripts, c.880C > T and c.3417dupG, showed read counts of 40% and 22%, respectively. The read counts for the samples containing the CTT transcripts, c.3873delC, c.3870_3879dup, c.3883C > T, and c.3903G > A, were 39.1%, 34.1%, 45%, and 52.7%, respectively (Fig. 3). With only two NTT samples, we were unable to demonstrate a significant reduction in gene expression of NTT transcripts compared to CTT with a lower read count identified only in N13.

### **Facial phenotyping using GestaltMatcher**

To objectively reproduce facial differences between the two *MN1* genotype subgroups, we used the GestaltMatcher approach to analyse 38 available facial images in the cohort. Facial similarities among individuals can be quantified using the cosine distance between representations in the CFPS (details in Methods).

**Fig. 4:**
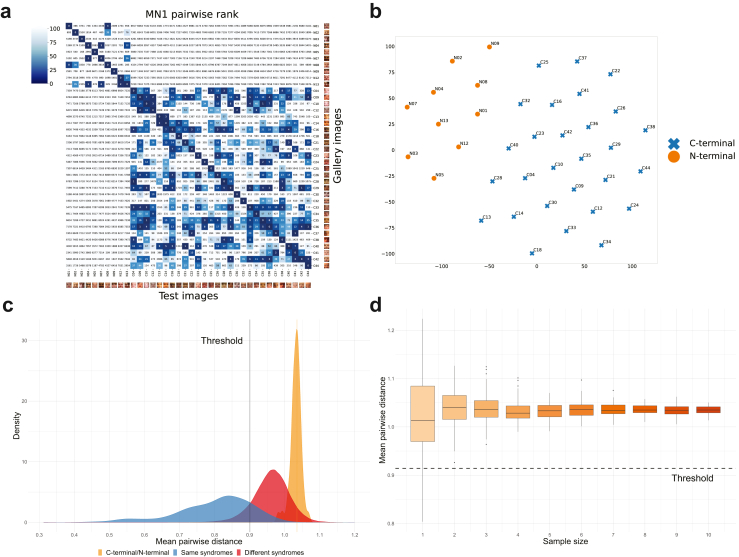
**GestaltMatcher AI-driven facial gestalt analysis. a**. The pairwise rank of 38 images (NTT:10 and CTT:28) in CFPS. Naming of photos with molecular location of expected truncation before patient ID. Gallery images were the images to be matched in CFPS. Each column is the result of testing one subject in the column and listing the rank of the other 37 photos in each row. For example, by testing C41 (the third column from the right), C42 was on the third rank, and C37 was on the first rank of C41. Both X and Y axes were sorted by the genomic location. The boundary of the NTT and the CTT separated the patients into two clear clusters. **b**. *t*-SNE visualization of Facial Phenotypic Descriptors of 38 images. Visualization of facial phenotypic descriptors by reduction down to two dimensions supports CTT (blue) and NTT (orange) as two distinct phenotypic entities. **c.** Comparing distance distribution of CTT and NTT patients to the distribution sampled from the same syndromes and different syndromes. The same syndrome distribution (blue) was sampled from the patients with the same syndrome, and the different syndrome distribution (red) was sampled from the patients with two different syndromes. 100% of the CTT and NTT distribution is above the threshold, indicating that patients with CTT and NTT present two different facial phenotypes. **d.** Downsampling analysis between the CTT and NTT groups. The X-axis is the sample included in each group. When both groups had at least two patients, the mean pairwise distance distribution was above the threshold.

We obtained the pairwise rank matrix for 38 images of the MN1 cohort along with 7459 of those from 5995 distinct subjects and reflecting 449 various disorders from the GestaltMatcher Database (GMDB)^49^ to visualize the positioning of individuals within the CFPS. As shown in Fig. 4a, NTT and CTT patients are separated into two distinct clusters, with a high degree of facial dysmorphic similarities between patients in the same cluster. We performed t-SNE^51^ (Fig. 4b) to visualize the distribution of patients in a two-dimensional space, which confirmed the separation of the CTT and NTT groups into two clusters. These results support the distinction of facial phenotypes between individuals with CTTs and NTTs as reported previously.^38^^,^^39^

### Statistical quantification of splitting in *MN1* using “GestaltMatcher lumping and splitting approach”

To determine the effectiveness of GestaltMatcher at distinguishing subgroups of syndromes due to variation within the same gene, we developed a specific approach designated the “GestaltMatcher lumping and splitting approach,” which is a statistical method centred around mean pairwise cosine distance in the CFPS. We selected 1499 images from 1182 distinct patients in the GMDB representing 321 various syndromes. Out of these, we sampled two control distributions: (1) a distribution of the pairwise distance between randomly sampled batches of patients with the same syndrome and (2) a distribution of the pairwise distance between batches of patients randomly sampled from two different syndromes. Following receiver operating characteristic (ROC) analysis, we defined a threshold (c = 0.901) to classify groups representing two different disorders with a sensitivity of 0.911 and a specificity of 0.885 on the validation fold (black vertical line in Fig. 4c. Details and results on remaining folds in Methods and Supplementary Table S2, Supplementary Fig. S2). We then compared the mean pairwise distance between the CTT (C) and NTT (N) groups in the two distributions, resulting in d(C,N)=1.035 (indicated by the orange vertical line in Fig. 4c) corresponding to a positive predictive value (PPV) of the cohorts being distinct of 96% (Supplementary Table S5. Since d(C,N)>c, this statistical method provided evidence that patients from the two groups expressed different facial phenotypes. To understand the uncertainty and consider the variances within each group, we sampled 100 subgroups of random size from the CTT and NTT groups and calculated the mean pairwise cosine distance. As shown in Fig. 4c, 100% of the sampled mean pairwise cosine distances between the subgroups were above the threshold, which provides further evidence of two different facial phenotypes based on a more robust statistical evaluation.

To further analyse the applicability of our method for assessing the stability of outcomes based on sample size, we sampled 1≤n≤min(|C|,|N|) patients from each group and calculated the mean pairwise cosine distance. For each n, samples were randomly drawn 100 times (Fig. 4d). The median pairwise distance was relatively stable within the samples: however, the variance decreased substantially with increasing sample size. This indicates that our method yields results with less uncertainty for larger cohorts; however, the two groups can still be separated with only one patient in each group.

### Distinct DNAm signatures for CTT and NTT variants

Peripheral blood DNA samples were available from ten individuals with CTT and five with NTT, of which two samples in the latter group were derived from a mother (N13) and her son (N12). Nine CTT samples were selected as a discovery cohort to outline the DNAm signature, whereas the remaining CTT sample and five NTT samples were used as the validation and testing datasets, respectively. Following DNAm analysis of peripheral blood using Infinium EPIC arrays and filtering the polymorphic single-nucleotide polymorphisms and nonspecific probes, 639,563 CpG sites were quantified using beta scores, which calculated DNAm levels as a ratio between 0 and 1 using the *minfi* Bioconductor package in *R*.

**Fig. 5:**
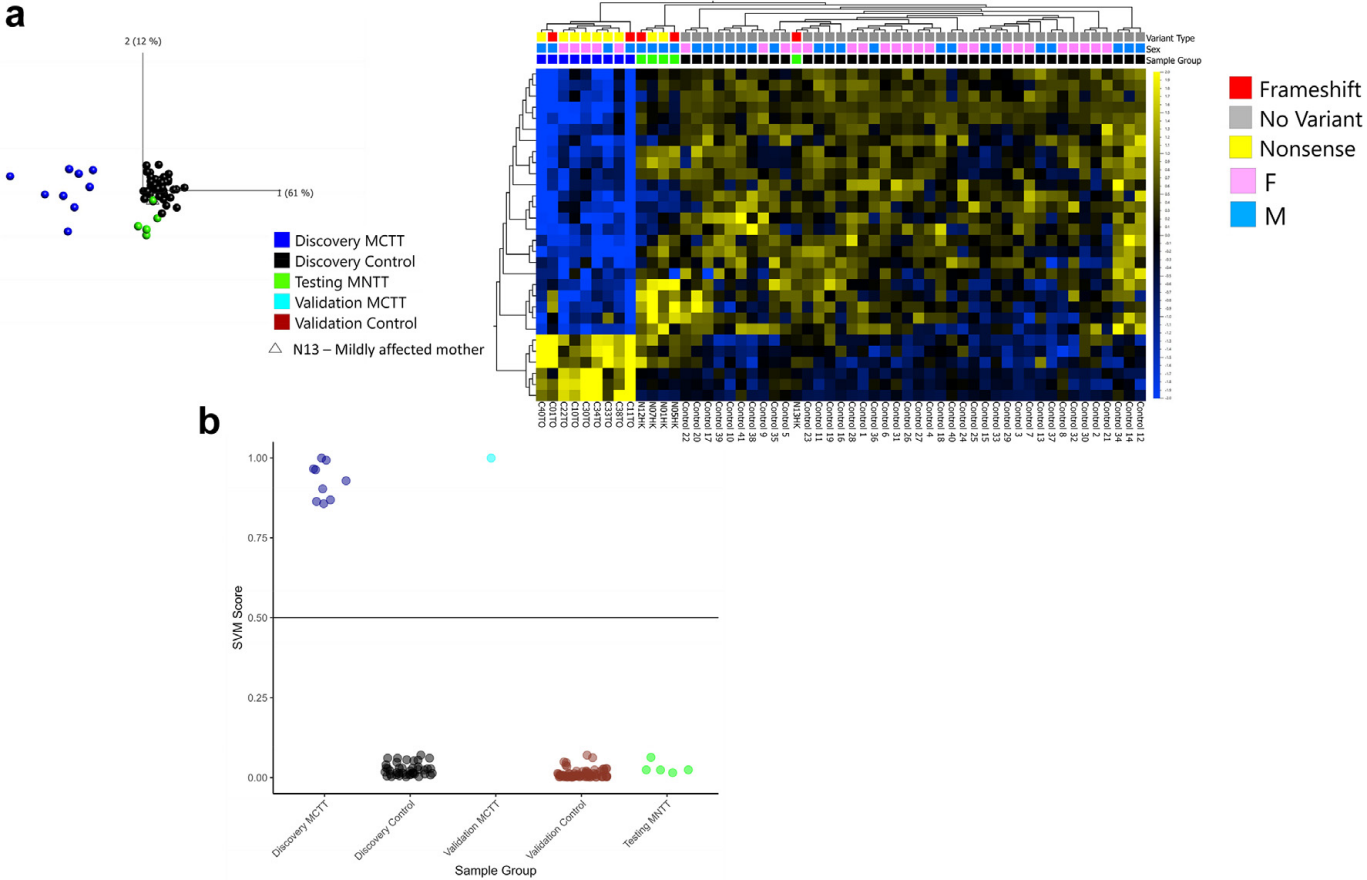
**DNAm signatures for truncating variants in *MN1.* a**. Principal component analysis (left) showing the separation between individuals with CTT (blue circles) and controls (black circles) from the discovery cohort at the CTT specific DNAm signature (30 CpG sites). Hierarchical clustering and heatmap (right) displaying the methylation profile between the 2 groups at the signature sites and the clustering of CTT individuals separate from controls. NTT *MN1* samples (green) separate from CTT (blue) with the affected mother (triangle, N13) clustering with controls. Percentage variation from principal component analysis in brackets. **b**. Classification of samples using SVM machine learning models based on each DNAm signature, showing a clear separation of CTT samples (dark blue) from NTT samples (green), thus validating the distinction of CTT and NTT samples by PCA plot. Scores closer to 1 indicate that the DNAm profile is positive and likely disease causing and scores closer to 0 indicate that the classification is negative and that the DNAm profile is more similar to controls.

We identified a DNAm signature for 30 differentially methylated CpG sites (q ≤ 0.05, |Δβ| ≥ 0.05) for CTT variants (i.e., for MCTT syndrome) after considering the covariates (i.e., age, blood cell counts, and sex) comparing to 41 age- and sex-matched control subjects (Fig. 5a). The 30 differentially methylated CpGs along with their genes and related functions are shown in Supplementary Table S6.

To classify these DNAm signatures, we created a support vector machine (SVM) training model using DNAm data from nine CTT variant samples and 41 controls using the CTT-specific signature (Fig. 5b). One CTT variant, five NTT variants, and an additional control cohort (n = 79) were used to validate and test the models. While there were insufficient number of NTT case samples to determine a definitive distinction of NTT with controls, the relative clustering of NTT samples suggests that an NTT DNAm signature may be identified with a larger sample size (Fig. 5a).

### Combining GestaltMatcher with DNAm to enhance the method of splitting other syndromes

To further examine the utility of GestaltMatcher for splitting by facial phenotype, we validated our approach on three other genes using data for known subsyndromes, each of which was previously characterized by methylation signatures (*SRCAP*,^30^ *SMARCA2*,^34^ and *ADNP*^45^) (Fig. 1). The number of patient photos used for GestaltMatcher for each gene, along with the corresponding analysis results, is listed in Supplementary Table S5. Three different subgroups representing *SRCAP* mutations and associated syndromes have been reported: Floating-Harbour syndrome (FLHS; OMIM #136140) with a clear episignature, proximal variants with developmental delay, hypotonia, musculoskeletal defects, and behavioural abnormalities (DEHMBA; OMIM #619595), and distal variants with DEHMBA. The latter two groups are considered clinically similar with a positive episignature identified only in 2 out of 5 of the patients in the distal group.^30^ All three SRCAP comparisons using GestaltMatcher suggested splitting the subgroups. The two comparisons of FLHS and the proximal and distal regions were both within the sampled distribution above the threshold (Supplementary Figs. S3 and S4, Supplementary Table S5) and the results were consistent with the different episignatures demonstrated in a previous study.^30^ Interestingly, 91.84% of the comparisons between individuals with proximal and distal variants were above the threshold (Supplementary Fig. S5, Supplementary Table S5); however, the published episignatures between these groups were not significantly different. The discrepancy between these parameters may be caused by the small sample size, because the distal group tested here consisted of only five individuals. More individuals in this group are required for further analysis, although we did not observe any bias for age or ethnic background between the distal and proximal groups. In the second example, variants in *SMARCA2* cause two disorders, Nicolaides–Baraitser syndrome (NCBRS; OMIM #601358) and Blepharophimosis-impaired intellectual development syndrome (BIS; OMIM #619293). The comparison between 77 NCBRS images and 30 representing BIS revealed a PPV of 96.64% and that 100% of the sampled distribution was above the threshold (Supplementary Fig. S6, Supplementary Table S5). Finally, a previous study showed that variants in *ADNP* were linked to two molecularly distinct DNAm signatures (ADNP-1 and ADNP-2).^45^ Testing facial images from these two subtypes (ADNP-1 with six images and ADNP-2 with seven images) revealed a PPV of 80.75% and that 99% of the comparisons were above the threshold (Supplementary Fig. S7, Supplementary Table S5). Overall, the subgroups could be split even with a limited number of individuals after downsampling (Supplementary Figs. S3c, S4c, S5c, S6c, and S7c). Therefore, GestaltMatcher analysis proved its ability to split clinical subgroups caused by variants in the same gene, which complies with the known different methylation signatures analysed in previous studies.

## Discussion

In this study, we proposed an AI-driven framework that combines NGP (GestaltMatcher) and DNAm analysis to address the challenges of syndrome delineation, particularly in distinguishing subgroups within a single gene. We used an extended cohort of 56 (22 unreported) individuals with *MN1* truncating variants to effectively demonstrate the utility of AI-driven approaches for distinguishing individuals with CTT variants (n = 43) from those with NTT (n = 13) and controls.

GestaltMatcher uses unsupervised learning to quantify facial dysmorphic features and performs statistical comparisons between subgroups without requiring pre-training for specific syndromes, making it suitable for ultra-rare disorders. To validate this framework, we applied five-fold cross-validation, achieving a sensitivity of 91.1% and specificity of 88.5% in distinguishing syndrome subgroups by facial analysis. For DNAm analysis, we employed a supervised Support Vector Machine (SVM) model trained and validated using internal cross-validation on methylation profiles to distinguish subgroups with distinct epigenetic signatures.

Our model was further validated by external datasets on syndromes with known DNAm signatures (e.g., *SRCAP*, *SMARCA2*, and *ADNP*), demonstrating generalizability and scalability. By enabling the early distinction of subgroups and facilitating informed lumping or splitting decisions, this framework paves the way for timely, accurate diagnoses and treatments, ultimately improving patient outcomes.

In the current genomic era, in which the discovery of novel phenotypes with a known molecular basis has grown by 257% from 2018 to 2021,^52^ and majority are neurodevelopmental syndromes.^53^ Although clinical or phenotypic features have traditionally been used to split diseases into distinguishable entities, these features are often nonspecific and may be common to different underlying molecular mechanisms. The complexity of dealing with pleiotropism and heterogeneity of genetic mechanisms at a given locus is a central issue that needs to be addressed with more accuracy. For the example of *MN1*, we hypothesized that differences in NMD escape between patients with NTT and CTT variants would correlate with the clinical differences between these groups. However, confirmation proved challenging as RNA-seq analysis was limited by low *MN1* expression in whole blood and the scarcity of fibroblast samples, and the results obtained suggested no consistent difference in transcript levels between the groups. As an alternate means of validating the *MN1* subgroup differences, we tested facial analysis by GestaltMatcher and DNAm profiling. This pipeline may be of value in lumper-splitter curations, led by the ClinGen working group, at loci where less obvious clinical subgroup distinctions can be made.

The framework provided by the ClinGen working group on lumping and splitting involves the following four levels of evidence: 1) (curated) assertion, 2) molecular mechanism, 3) phenotypic variability, and 4) inheritance pattern.^44^ Although some gene-disease curations can be split based on differences in inheritance patterns, other genes require a detailed characterization of molecular mechanisms and phenotypic variability. Based on the ClinGen working group, there is a general preference to lump assertions within the same gene unless there is a clear indication of splitting, whereas the indications to lumping include the following: i) an assentation for only one disease entity shown in previous reports ii) molecular mechanisms involved in disease entities that do not differ iii) intrafamilial phenotypic variability is more prominent compared with interfamilial variability, iv) difference in the inheritance pattern for the disease entities is reflected in a continuum of disease, and v) disease entities compose a part of a variable phenotype detected within a single organ system and there is not enough evidence for a single phenotype.

In our DNAm experiments, we demonstrated the power of episignatures in detecting a unique MCTT signature. The signature CpG sites not only help in separating individuals with CTT from the normal individuals for a functional molecular diagnosis, but also in differentiating from individuals with NTT who do not share the same DNAm pattern as those with CTT. Individuals with NTT and CTT exhibit strikingly distinct craniofacial phenotypes, and neurodevelopmental defects are more severe for those with CTT (Table 1). A conclusive clinical diagnosis for these *MN1* variant subgroups is therefore relatively easy, but diagnosis for other *MN1* variant types (e.g., missense) associated with phenotypes only partly overlapping those already described may be more problematic. Episignatures provide a functional molecular validation for these potential cases with clinical overlap, as previously demonstrated in a study that developed a SVM model on unique DNAm signatures of phenotypically overlapping CHARGE and Kabuki syndromes.^54^ This model enabled the highly specific and sensitive prediction of pathogenicity, which would otherwise be misclassified by clinical diagnostic criteria. The distinct DNAm profiles successfully classified these complicated cases into different genetic aetiologies with overlapping phenotypes, providing supportive evidence for characterizing the underlying molecular mechanisms. Moreover, episignature-based machine learning models have the capability of classifying gain-of-function variants from the loss-of-function variants within the same gene,^55^ highlighting the potential of this computational tool to distinguish variants with opposite disease mechanisms. In this study, the PCA plots and SVM models derived from the DNAm signatures of CTT patients demonstrated that the described signatures were not identified in the NTT patients, supporting our previous hypothesis of alternative molecular mechanisms within the *MN1* cohort.^41^^,^^42^ It should be noted that these DNAm profile analyses were based on blood-derived signatures, which may overlap with differentially expressed genes from biologically relevant tissues such as neurons.^27^ As most epigenetic regulators are ubiquitously expressed and DNAm patterns established from early development can be maintained life-long, episignatures can be identified across cell types, enabling the investigation of molecular mechanisms from clinically accessible tissues. DNAm signature profiling is therefore a feasible and clinically accessible tool for providing supportive evidence for molecular mechanisms.

A recognizable facial gestalt has been previously described in individuals with CTT variants, but the demarcation of facial features between patients with NTTs and controls has been less evident. GestaltMatcher confirmed the distinction between the facial features of individuals affected by NTTs and CTTs (and between NTTs and controls; see the distinctiveness of the NTT subgroup section in Supplementary Materials). We proposed a statistical threshold to objectively quantify this demarcation by elaborating on the original GestaltMatcher algorithm. With the availability of frontal images of the face, this method could be easily applied to the diagnosis of any syndrome involving craniofacial anomalies. The added advantage is that the GestaltMatcher method can be implemented with only a few images (minimum of three) without prior training of the photos in the model. We propose this technique as a first-line tool to prioritize and strengthen the evidence for phenotypic variability. Subtle differences in facial gestalt and clinical phenotype are often less identifiable by the human eye and the low number of photos necessary will be advantageous in ultrarare and novel cases. We also demonstrated that GestaltMatcher was effective for splitting subgroups within the same gene, such as FLHS and proximal or distal mutation groups in the *SRCAP* gene, NCBRS and BIS in *SMARCA2*, and two subtypes of *ADNP*. Thus, the tool can streamline resources for the functional validation of gene–disease associations that are more resource-intensive. Moreover, it can further facilitate curation in the ClinGen lumping and splitting working group.^56^

We acknowledged several limitations of our methods. For GestaltMatcher, the accuracy of the tool may be affected by variations in age and ethnic background. In the future, potential bias due to sex and ethnicity may be further compensated by unlearning the bias during model training or using a more diverse training dataset.^57^ As this is an ultra-rare disease, the sample size is limited by the number of available cases recruited, yet we aim to demonstrate that these tools can help with splitting decisions despite a low number of samples. The lack of significant difference in *MN1* expression in fibroblasts following RNA-seq of NTT and CTT subgroups needs to be confirmed with further samples. The tissue type used for RNA-seq may have had a minor influence on the results, which could be addressed by trans-differentiation of patient fibroblasts into other disease-relevant cell types e.g., osteoblasts. Nevertheless, efficiency of NMD is not expected to have significant variations among expressed tissues and hence this was not pursued. Given the lack of fibroblast samples, we established blood DNAm profiling as an alternative tool for molecular phenotyping in *MN1* patients.

In conclusion, our detailed analysis performed in an expanded *MN1* cohort demonstrated the effectiveness of DNAm and GestaltMatcher in providing significant evidence for the splitting of a neurodevelopmental syndrome into multiple dimensions, which will be helpful as an adjunct in the early stages of syndrome discovery and may be used for interpreting splitting decisions in novel and ultrarare neurodevelopmental syndromes.

## Contributors

C.C.Y.M. and T.H. conceptualized and designed the study, while T.H., B.Chung and R.W. supervised the project. The clinician scientists E.P., F.L., J.D., P.A.S, H.M.B., J.A.B., L.F., F.T.M, I.V.P, N.C., A.S., Y.Z., B.Callewaert, E.B.O., A.J., A.Z., M.W., L.D., B.I., B.Cogne, O.B., C.V., A.G., E.S., K.L., S.K., V.G., J.A., A.L., C.G., D.D. and B.Chung recruited the patients, provided relevant clinical data, biological samples and analysed the data. C.C.Y.M, H.K., T.H., P.K., S.C., N.R., A.K.C., M.C., M.L. and D.D. performed the analysis and both C.C.Y.M and T.H. have accessed and verified the data reported. C.C.Y.M, T.H., H.K., S.C., A.L. and C.G. were involved in writing up and critically reviewing the manuscript. All authors read and approved the final version of the manuscript.

## Data sharing statement

Clinical and genetic data described in this study are included in the additional files. The code for GestaltMatcher statistical analysis is available at https://github.com/igsb/GestaltMatcher/tree/eBioMedicine. The frontal images are available from GestaltMatcher Database (https://db.gestaltmatcher.org/) with restrictions due to reasons of sensitivity. Access will be granted upon the submission of a request reviewed by the committees. The DNAm and RNAseq datasets supporting the current study have not been deposited in a public repository due to institutional ethics restrictions. The analytical pipeline can be accessed through EpigenCentral at https://epigen.ccm.sickkids.ca. All R-packages used in the study are publicly available as described in the methods section. Principal component analysis and the heatmaps were generated using Qlucore Omics Explorer 3.8.

## Declaration of interests

The authors declare that they have no competing interests.
