# Supplementary material for: Artificial intelligence-driven genotype–epigenotype–phenotype approaches to resolve challenges in syndrome diagnostics
Source: eBioMedicine. 2025 Apr 24;115:105677. doi: 10.1016/j.ebiom.2025.105677 (PMC12242594; doi:10.1016/j.ebiom.2025.105677)
Supplement: Supplementary Materials [file mmc7.docx]

**Artificial intelligence-driven genotype–epigenotype–phenotype approaches to resolve challenges in syndrome diagnostics**

**Supplementary Material**

Christopher C.Y. Mak^1,35^, Hannah Klinkhammer^2,3,35^, Sanaa Choufani^4,35^, Nikola Reko^4^, Angela Christman^5^, Elise Pisan^6^, Martin M.C. Chui^1^, Mianne Lee^1^, Fiona Leduc^7^, Jennifer C. Dempsey^5^, Pedro A. Sanchez-Lara^8^, Hannah M. Bombei^9^, John A. Bernat^9^, Laurence Faivre^10,11^, Frederic Tran Mau-Them^10,12^, Irene Valenzuela Palafoll^13^, Natalie Canham^14^, Ajoy Sarkar^15^, Yuri A. Zarate^16,17^, Bert Callewaert^18^, Ewelina Bukowska-Olech^19^, Aleksander Jamsheer^19,20^, Andreas Zankl^21,22^, Marjolaine Willems^23^, Laura Duncan^24^, Bertrand Isidor^25^, Benjamin Cogne^26^, Odile Boute^7^, Clémence Vanlerberghe^7^, Alice Goldenberg^27^, Elliot Stolerman^28^, Karen Low^29,30^, Vianney Gilard^31^, Jeanne Amiel^6^, Angela E. Lin^32^, Christopher T. Gordon^6^, Dan Doherty^5^, Peter M. Krawitz^2^, Rosanna Weksberg*^33,34,36^, Tzung-Chien Hsieh*^2,36^, Brian H.Y. Chung*^1,36^

^1^Department of Paediatrics and Adolescent Medicine, School of Clinical Medicine, The University of Hong Kong, Hong Kong SAR, China.
^2^Institute for Genomic Statistics and Bioinformatics, University Hospital Bonn, Rheinische Friedrich-Wilhelms-Universität Bonn, Bonn, Germany.
^3^Institute for Medical Biometry, Informatics and Epidemiology, University Hospital Bonn, Rheinische Friedrich-Wilhelms-Universität Bonn, Bonn, Germany
^4^Genetics and Genome Biology Program, Research Institute, the Hospital for Sick Children, Toronto, ON M5G 1X8, Canada.
^5^Department of Pediatrics, University of Washington, Seattle, WA 98195, USA
^6^Laboratory of Embryology and Genetics of Human Malformations, Institut National de la Santé et de la Recherche Médicale (INSERM) UMR 1163, Institut Imagine, Université Paris Cité, 75015 Paris, France
^7^CHU Lille, Centre de référence Anomalies du développement et syndromes malformatifs, F-59000 Lille, France
^8^Department of Pediatrics, Cedars-Sinai Medical Center, Los Angeles, California, USA.
^9^Division of Medical Genetics and Genomics, Stead Family Department of Pediatrics, University of Iowa Hospitals, Iowa City, Iowa, USA.
^10^Centre de Génétique et Centre de Référence Anomalies du Développement et Syndromes Malformatifs, FHU TRANSLAD, Institut GIMI, Hôpital d’Enfants, CHU Dijon-Bourgogne, Dijon, France
^11^Equipe GAD INSERM UMR1231, Université de Bourgogne Franche Comté, Dijon, France
^12^UF 6254 Innovation en diagnostic génomique des maladies rares, Centre Hospitalier Universitaire de Dijon, Dijon, France
^13^Department of Clinical and Molecular Genetics, University Hospital Vall d´Hebron and Medicine Genetics Group, Valle Hebron Research Institute, Barcelona, Spain.
^14^Liverpool Centre for Genomic Medicine, Liverpool Women's Hospital, Crown Street, Liverpool, UK.
^15^Department of Clinical Genetics, Nottingham University Hospitals National Health Service Trust, Nottingham NG5 1PB, UK.
^16^Section of Genetics and Metabolism, University of Arkansas for Medical Sciences, Little Rock, AR 72701, USA.
^17^Division of Genetics and Metabolism, University of Kentucky, Lexington, KY
^18^Center for Medical Genetics Ghent, Ghent University Hospital, Ghent 9000, Belgium; Department of Biomolecular Medicine, Ghent University, Ghent 9000, Belgium.
^19^Department of Laboratory Diagnostics, Poznan University of Medical Sciences, Poznan, Poland.
^20^Centers for Medical Genetics GENESIS, Poznan, Poland
^21^Department of Clinical Genetics, The Children's Hospital at Westmead, Sydney, Australia
^22^Faculty of Medicine and Health, The University of Sydney, Sydney, Australia; Garvan Institute of Medical Research, Sydney, Australia
^23^Unité INSERM U 1051, Département de Génétique Médicale, CHRU de Montpellier, Montpellier, France.
^24^Department of Medicine, Vanderbilt University Medical Center, Nashville, Tennessee.
^25^Service de Génétique Médicale and L'institut du Thorax, CHU Nantes, Nantes Université, CNRS, INSERM, Nantes, France.
^26^Medical Genetics Service, Nantes University Hospital Center, Nantes, France.
^27^Normandie Univ, UNIROUEN, Inserm U1245, CHU Rouen, Department of Genetics and Reference Center for Developmental Disorders, FHU G4 Génomique, F-76000, Rouen, France.

^28^Greenwood Genetic Center, South Carolina, USA

^29^Centre for academic child health, Bristol Medical School, University of Bristol, UK

^30^Department of clinical genetics, UHBW NHS trust, Bristol, UK
^31^Department of Pediatric Neurosurgery, Rouen University Hospital, 76000 Rouen, France.
^32^Medical Genetics, Mass General for Children, Boston, MA 02114, USA
^33^Genetics and Genome Biology Program, Research Institute, the Hospital for Sick Children, Toronto, ON M5G 1X8, Canada
^34^Division of Clinical and Metabolic Genetics, Department of Pediatrics, the Hospital for Sick Children, University of Toronto, Toronto, ON M5G 1X8, Canada

^35^These authors contributed equally: Christopher C.Y. Mak, Hannah Klinkhammer, Sanaa Choufani

^36^These authors jointly supervised this work: Rosanna Weksberg, Tzung-Chien Hsieh*, Brian H.Y. Chung*

*Corresponding Authors: Tzung-Chien Hsieh (Tel: +49 228-287-13533; email: thsieh@uni-bonn.de), Brian H.Y. Chung (Tel: +852 2255-4482; email: bhychung@hku.hk)

**GestaltMatcher gestalt validation approach (assessing the similarity within one group)**

To test whether individuals within one group were likely to share a common phenotype, we compared them with random patients from the GMDB. A control distribution was constructed reflecting the mean pairwise distance within one group. Using the same data as above (i.e., 1,499 images from 1,182 subjects and 321 syndromes), we sampled a distribution of mean pairwise distances in individuals with several different syndromes. We sampled 3,586 groups of random sample size from all patients. Next, we calculated the mean pairwise distance within the group of patients with an NTT variant and compared it with the control distribution.

**Distinctiveness of the NTT subgroup**

To determine whether patients of the NTT subgroup were phenotypically similar and formed an associated cluster, we used a different approach to simulate an NTT vs. control analysis by comparing the NTT group with groups created from random patients. We simulated a control distribution of the pairwise distance between batches of patients randomly sampled from the GMDB, i.e. patients with different syndromes. The mean pairwise distance within the NTT subgroup was $d\left( N \right)=0.88$ and therefore falls into the lowest 0.1% of the control distribution of mean pairwise distances among random patients. Thus, the gestalt validation analysis provided evidence that patients within the NTT subgroups show phenotypic similarity as a group and are, therefore, distinct from the control population.


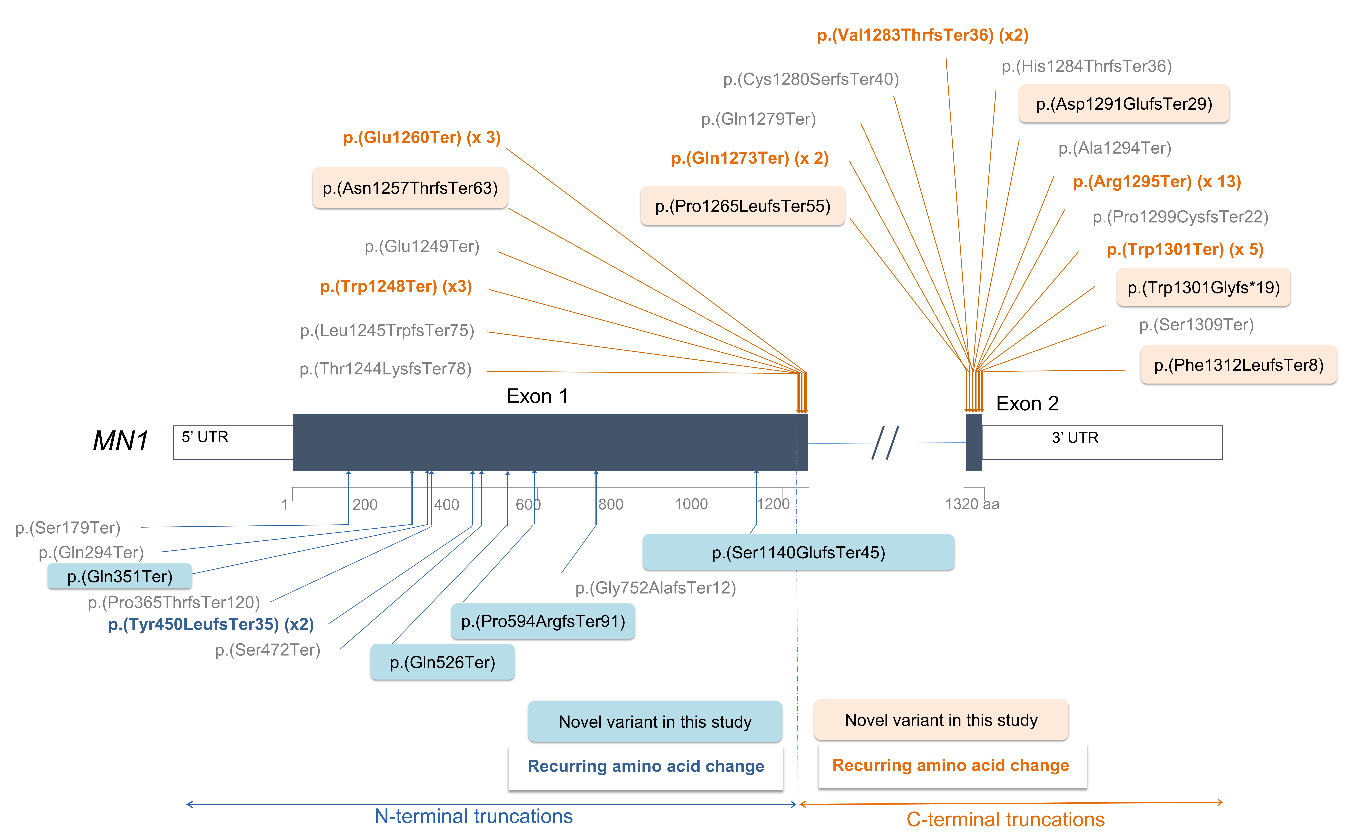


**Figure S1 | Mutation topology and predicted boundary of NTT and CTT variants in the *MN1* gene.** Topological representation of all reported variants in the two exons of *MN1* gene. Novel variants are highlighted with boxes and recurrent variants are shown in bold. Recurrent protein changes are indicated by “x” followed by the number of recurrences. The delineation of N-terminal truncation (NTT) and C-terminal truncation (CTT) variants are determined by the expected position of the transcript escaping nonsense mediated decay (NMD) at amino acid position 1244.  


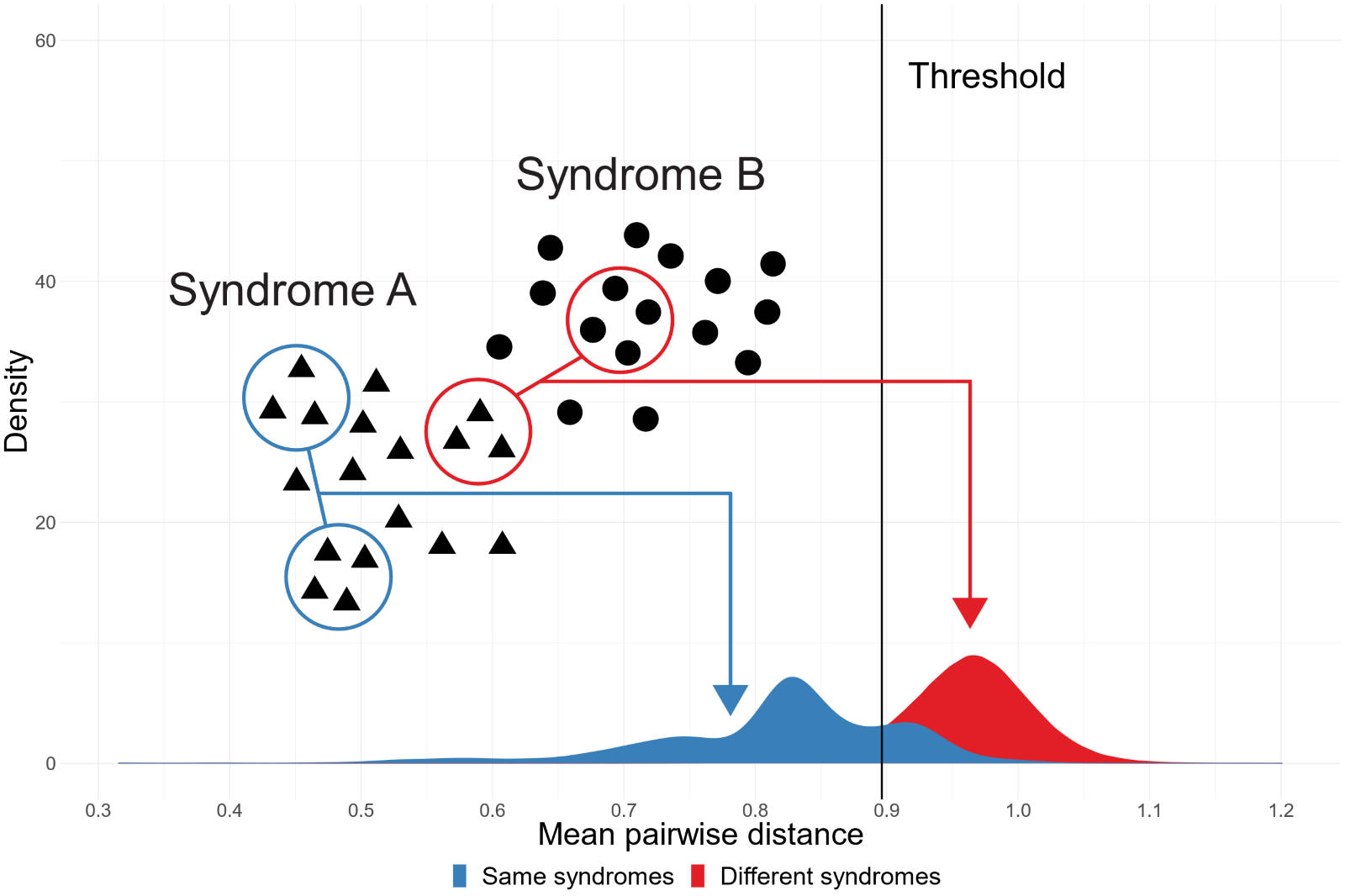


**Figure S2 | Concept of GestaltMatcher lumping and splitting analysis to build distributions of same and different syndromes**. Syndrome A (triangle) and syndrome B (dot) were randomly selected from 321 different syndromes. We then randomly selected two subgroups in syndrome A to calculate the mean pairwise distance between these two subgroups for the distribution of same syndrome (blue distribution). On the other hand, we randomly selected one subgroup from syndrome A and another subgroup from syndrome B, and calculated the mean pairwise distance between these two subgroups to build the distribution of different syndromes (red distribution). For each combination of syndrome A and syndrome B, we performed 100 random samplings.


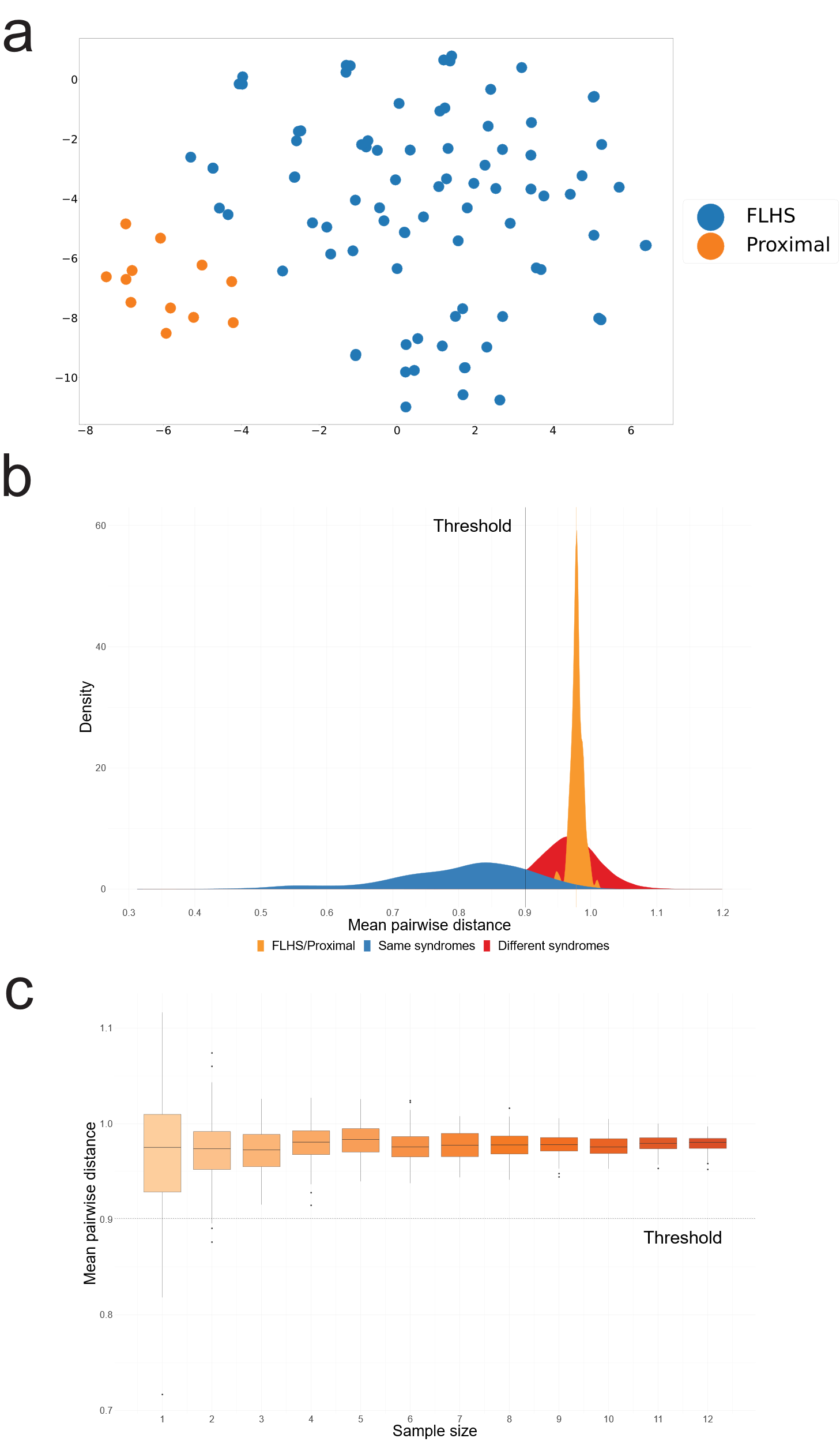


**Figure S3 |Results of splitting analysis between FLHS and Proximal in *SRCAP*.** a) t-SNE plot; b) distribution plot of comparisons of FLHS and Proximal groups; c) downsampling analysis between FLHS and Proximal groups. FLHS is Floating-Harbor syndrome (OMIM #136140). Proximal refers to the subgroup in Developmental delay, hypotonia, musculoskeletal defects, and behavioral abnormalities (DEHMBA; OMIM #619595).


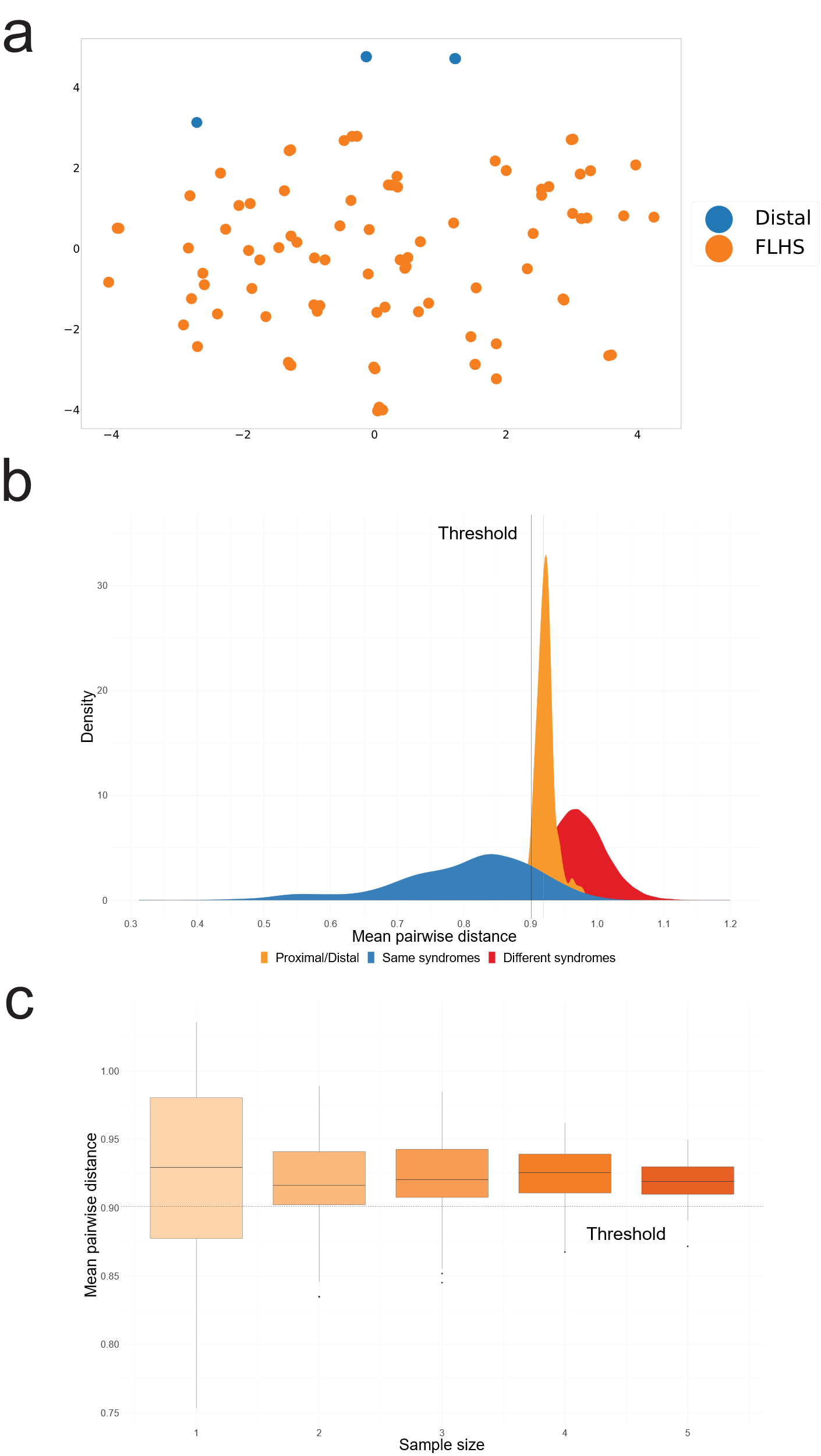


**Figure S4 |** **Results of splitting analysis between FLHS and Distal in *SRCAP*.** a) t-SNE plot; b) distribution plot of comparisons of FLHS and Distal groups; c) downsampling analysis between FLHS and Distal groups. FLHS is Floating-Harbor syndrome (OMIM #136140). Distal refers to the subgroup in Developmental delay, hypotonia, musculoskeletal defects, and behavioral abnormalities (DEHMBA; OMIM #619595). Five images from 3 patients were included in the distal group hence only three overlapping blue dots are shown.


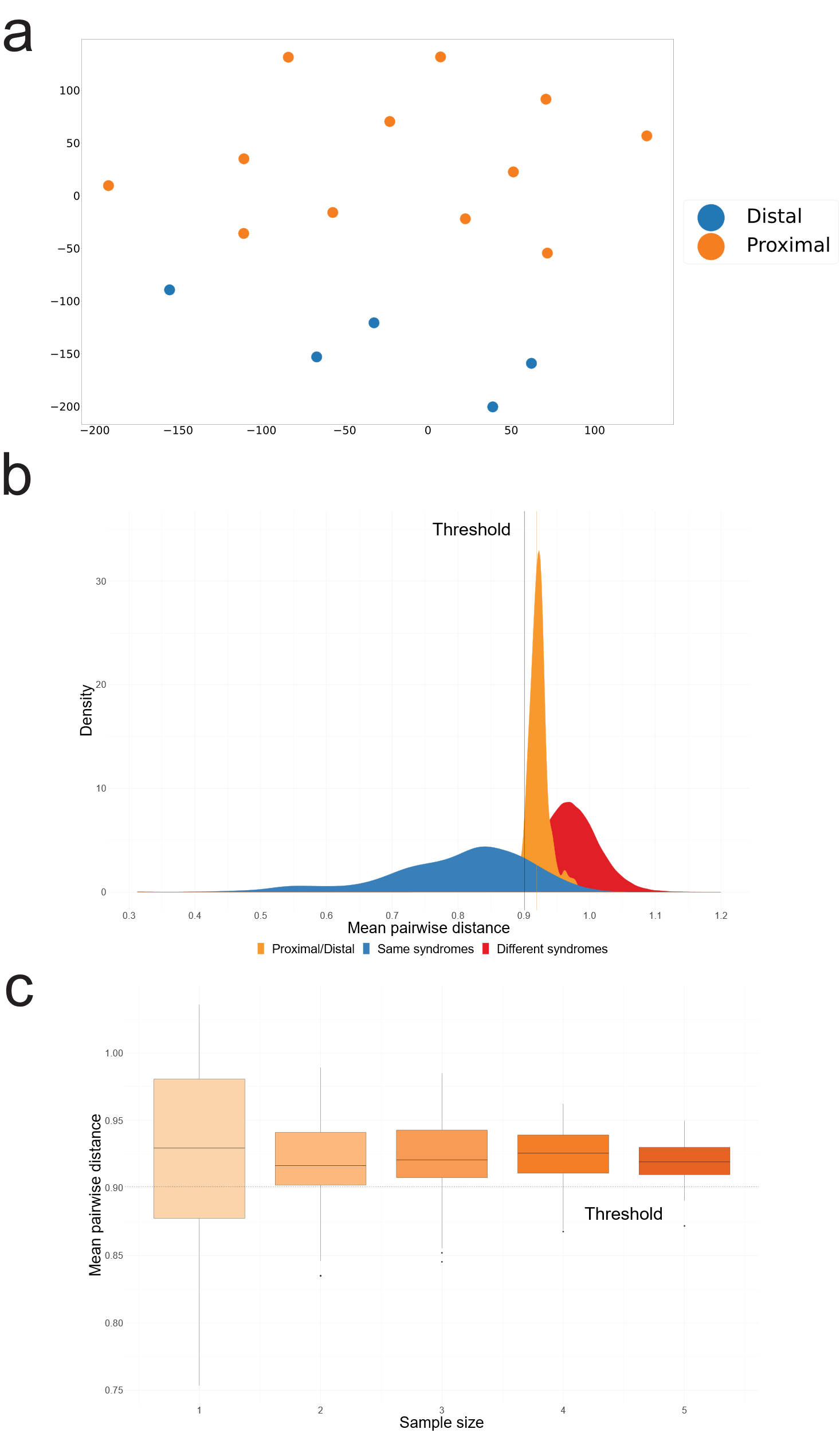


**Figure S5 |** R**esults of splitting analysis between Proximal and Distal DEHMBA groups in *SRCAP*.** a) t-SNE plot; b) distribution plot of comparisons of Proximal and Distal DEHMBA groups; c) downsampling analysis between Proximal and Distal DEHMBA groups.


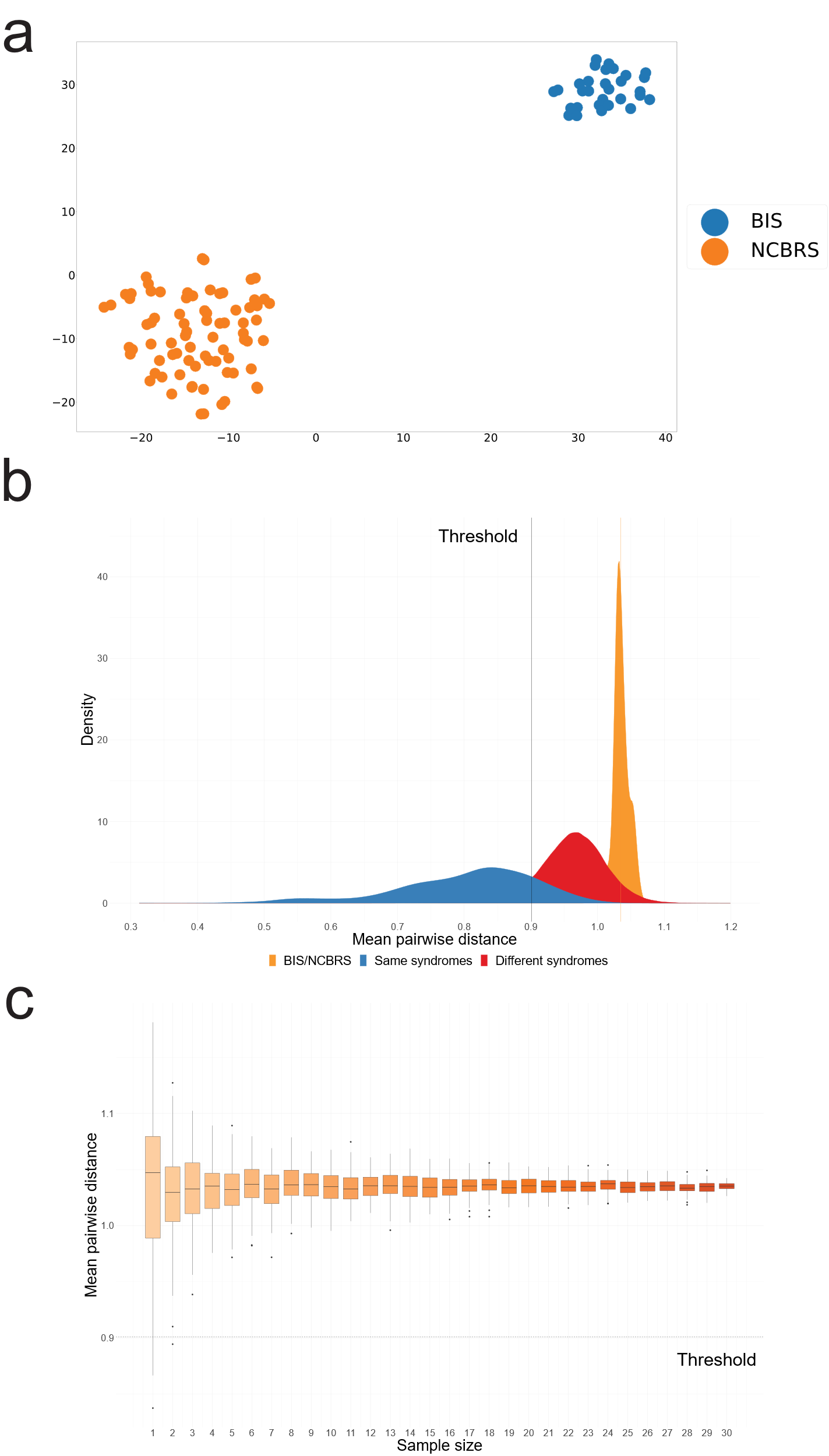


**Figure S6 |Results of splitting analysis between NCBRS and BIS in *SMARCA2*.** a) t-SNE plot; b) distribution plot of comparisons of NCBRS and BIS groups; c) downsampling analysis between NCBRS and BIS groups. NCBRS is Nicolaides-Baraitser syndrome (OMIM #601358). BIS is Blepharophimosis-impaired intellectual development syndrome (OMIM #619293).


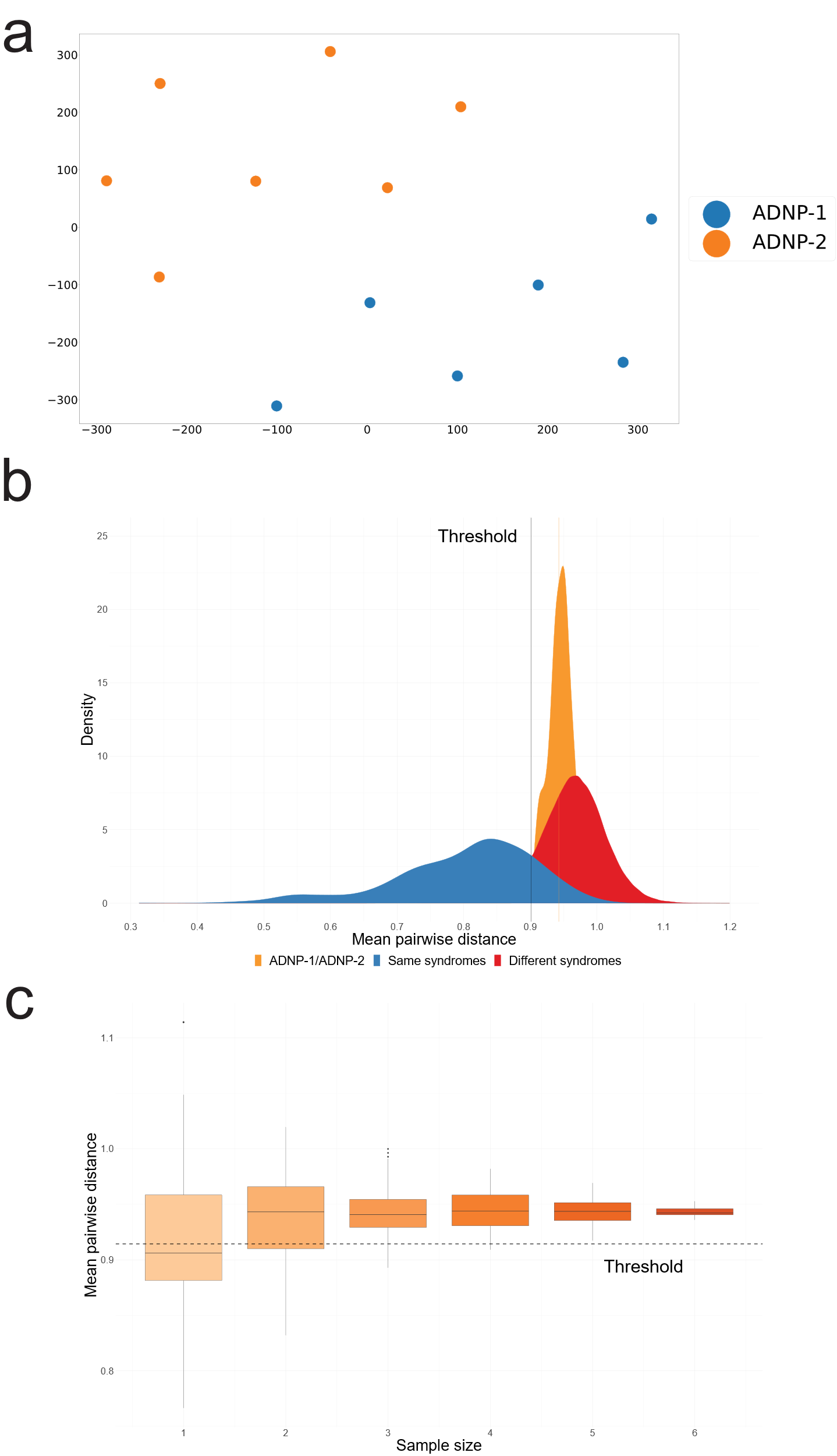


**Figure S7 |** **Results of splitting analysis between ADNP-1 and ADNP-2 in *ADNP*.** a) t-SNE plot; b) distribution plot of comparisons of ADNP-1 and ADNP-2 groups; c) downsampling analysis between ADNP-1 and ADNP-2 groups.
